# Influence of maternal zinc supplementation on the development of autism-associated behavioural and synaptic deficits in offspring *Shank3*-knockout mice

**DOI:** 10.1186/s13041-020-00650-0

**Published:** 2020-08-05

**Authors:** Yukti Vyas, Kevin Lee, Yewon Jung, Johanna M. Montgomery

**Affiliations:** Department of Physiology and Centre for Brain Research, Faculty of Medical and Health Sciences, University of Auckland, Private Bag 92019, Auckland, New Zealand

**Keywords:** Autism, Zinc, Shank3, Maternal diet, Glutamate, Synapse

## Abstract

Autism Spectrum Disorders (ASD) are characterised by deficits in social interactions and repetitive behaviours. Multiple ASD-associated mutations have been identified in the Shank family of proteins that play a critical role in the structure and plasticity of glutamatergic synapses, leading to impaired synapse function and the presentation of ASD-associated behavioural deficits in mice. Shank proteins are highly regulated by zinc, where zinc binds the Shank SAM domain to drive synaptic protein recruitment and synaptic maturation. Here we have examined the influence of maternal dietary zinc supplementation during pregnancy and lactation on the development of ASD-associated behavioural and synaptic changes in the offspring *Shank3* knockout (*Shank3*^*−/−*^) mice. Behavioural and electrophysiological experiments were performed in juvenile and adult *Shank3*^*−/−*^ and wildtype littermate control mice born from mothers fed control (30 ppm, ppm) or supplemented (150 ppm) dietary zinc. We observed that the supplemented maternal zinc diet prevented ASD-associated deficits in social interaction and normalised anxiety behaviours in *Shank3*^*−/−*^ offspring mice. These effects were maintained into adulthood. Repetitive grooming was also prevented in adult *Shank3*^*−/−*^ offspring mice. At the synaptic level, maternal zinc supplementation altered postsynaptic NMDA receptor-mediated currents and presynaptic function at glutamatergic synapses onto medium spiny neurons in the cortico-striatal pathway of the *Shank3*^*−/−*^ offspring mice. These data show that increased maternal dietary zinc during pregnancy and lactation can alter the development of ASD-associated changes at the synaptic and the behavioural levels, and that zinc supplementation from the beginning of brain development can prevent ASD-associated deficits in *Shank3*^*−/−*^ mice long term.

## Introduction

Autism Spectrum Disorders (ASDs) are clinically diagnosed by the presence of behavioural symptoms in two core criteria: A) impairments in social communication, and B) restricted and repetitive behaviours [3]. Patients with ASD also present non-diagnostic yet well-documented symptoms such as heightened anxiety, sensory sensitivity and altered sensory processing, changes in trace element metabolism, gut dysbiosis, and cognitive deficits [9, 35, 47, 54].

ASD-associated genetic alterations have been identified at many levels in the human genome. When functionally grouped, many ASD-associated genetic mutations converge on specific biological pathways involved in neuronal connectivity and synaptic plasticity. The Shank family of synaptic proteins (Shank1–3) exhibit a high degree of ASD-associated point, frameshift, and deletion mutations. Shank proteins form supramolecular complexes within the glutamatergic postsynaptic density (PSD) by interacting with other PSD scaffolding proteins to regulate membrane receptors, intracellular signalling molecules and the dendritic cytoskeleton, and thus are known as “master regulators” of the synapse. Consequently, *Shank* mutations can result in severe synapse dysfunction. *SHANK1* gene mutations have been found at a significantly higher prevalence in ASD patients than in matched controls. However, it is not a causative mutation, and patients with *SHANK1* mutations and animal models with *Shank1* deletions display milder ASD phenotypes [31, 35, 56, 58, 60]. Similarly, mutations in *SHANK2* gene have also been identified in ASD patients, including missense variations, duplications, deletions and premature truncations in the *SHANK2* gene [7, 14, 39, 52, 55]. Some ASD-associated behavioural deficits are evident in rodent models with *Shank2* deletions, however opposing synaptic alterations have been demonstrated in these models [40, 51, 57, 68]. In comparison to *SHANK1* and *SHANK2* mutations, ASD-associated *SHANK3* mutations are highly penetrant and more extensively studied. Several lines of *Shank3* mutant mice have been created with germline deletions of *Shank3* exons encoding different Shank protein domains, however, these *Shank3*^*−/−*^ models consistently present deficits in social interactions and heightened self-grooming [10, 21, 33, 36, 50, 57, 65, 66, 70, 72, 73]. *Shank3*^*−/−*^ mice display glutamatergic synaptic structural changes including reduced levels of PSD proteins such as synaptic Homer and SAPAP, and reduced expression of AMPAR and NMDAR subunits [33, 50, 57, 65, 66, 73]. Shank3 is most abundantly expressed in the striatum, and electron microscopy examination of cortico-striatal synapses onto medium spiny neurons (MSNs) demonstrated reduced PSD thickness and length, and reduced spine density [50]. Functionally, *Shank3*^*−/−*^ mice display impaired striatal synaptic transmission [21, 33, 50, 65, 73], and reduced hippocampal CA1 synaptic transmission is evident in some *Shank3*^*−/−*^ mouse models [10, 41, 59, 70], but not in others [50, 66]. Overall, *Shank3* mutations negatively impact synapse physiology. The phenotypic variability evident between different *Shank3*^*−/−*^ mouse models could be attributable to the differences in *Shank3* exon(s) deleted, animal age at experiment, and sex examined. However, this variability is consistent with the clinical heterogeneity observed in patients with ASD-associated *SHANK3* mutations.

Shank protein function is not only affected by genetic mutations but also by the local microenvironment within the post-synapse, notably the concentration of zinc. Zinc is enriched within the post-synapse at a concentration greater than 4 nmol per gram of protein, where zinc can modulate PSD scaffolding proteins including Shank [32]. Approximately 90% of total brain zinc is protein-bound, and free zinc is stored in glutamatergic presynaptic vesicles and released as a co-transmitter with glutamate [22, 53]. In response to neuronal depolarisation, postsynaptic zinc levels increase transiently and reversibly [27]. Zinc plays a critical role in the recruitment, clustering, density and stability of Shank2 and Shank3 through the formation of multimeric sheets at the PSD [4, 6, 23, 61]. Additionally, zinc signalling through Shank2 and Shank3 shapes the biophysical properties of developing glutamatergic synapses by dynamically regulating the AMPAR subunit switch from GluA2-lacking to GluA2-containing AMPARs [27]. Therefore, through its interaction with Shank proteins, zinc indirectly influences the overall PSD structure and alters postsynaptic receptor expression, as well as coordinating increased presynaptic neurotransmitter release.

The incidence of zinc deficiency is up to 50% in children with ASD [19, 67, 69, 71]. Zinc deficiency affects several neurological functions and neuronal maturation, and is a risk factor of ASD. Prenatal zinc-deficient mice display characteristic ASD-behaviours [23, 24], and animal models of postnatal zinc deficiency have also shown impairments in learning and memory, social interactions deficits, impaired vocalisations, and enhanced anxiety in adulthood [28]. Therefore, zinc is critical in neurodevelopment and zinc deficiency/mismanagement is linked to the pathogenesis of ASD.

Dietary zinc supplementation in *Shank3*^*ex13–16−/−*^ mice for 6 weeks post-weaning has recently been shown to prevent ASD-associated repetitive behaviours as well as social deficits, and cause an increase in Shank2 expression at cortico-striatal synapses [21]. Thus zinc supplementation post-weaning can reverse ASD-associated behaviours and cause significant synaptic structural and functional changes. Here we have examined the effects of zinc supplementation from the beginning of brain development (that is, during pregnancy and lactation). Specifically, we have addressed whether maternal dietary zinc can prevent *Shank3*^*−/−*^ induced behavioural and synaptic deficits becoming evident in the juvenile mice pups, and subsequently, if any zinc-induced effects in these mice are maintained into adulthood. Our data show significant effects on both ASD-associated behaviours and on glutamatergic synaptic transmission in the cortico-striatal pathway in juvenile and adult *Shank3*^*−/−*^ mice, highlighting that maternal dietary zinc supplementation during pregnancy and lactation can alter neuronal function and ASD-associated behaviours in offspring.

## Methods and materials

### Animals

All animal manipulations and experiments were conducted under regulations approved by the University of Auckland Animal Ethics Committee, and in adherence to the ARRIVE guidelines. *Shank3*^*ex13–16−/−*^ (B6.129-*Shank3*^*tm2Gfng*^/J) mice [50] were imported from Jackson Laboratories, Bar Harbor, ME, USA and maintained at the University of Auckland, Auckland, New Zealand. Shank3-wildtype, heterozygous and homozygous mice were produced from heterozygous x heterozygous breeding pairs. The breeding and experimental mice were housed under a normal 12/12-h light-dark cycle. A maximum of six mice were housed in individually ventilated chamber (IVC) cages (Tecniplast GM500 Mouse IVC Green Line). Food and water were available ad libitum.

### Experimental animals

The animals manipulated in this study were fed either a control zinc diet (30 ppm zinc, Research Diets Inc. D19410B) or a zinc supplemented diet (150 ppm zinc, Research Diets Inc. D06041101). These egg white-based diets contain 20% protein, 68% carbohydrate and 12% fat, and are identical in composition except for their zinc levels (Supplementary Table 1). These dietary zinc levels were tolerable and non-toxic, and did not have adverse effects on animal health, development, or reproduction (Supplementary Figure 1, [21]). Shank3 heterozygous male and female mice were assigned to a control zinc diet (30 ppm) or a zinc supplemented diet (150 ppm) upon weaning at postnatal day 21 (P21). At 6 weeks of age, one male and one female heterozygous mouse on the same zinc diet were housed together for breeding. Their pups were ear-punched for genotyping and identification at postnatal day 10 (P10). The tissue collected from each ear-punch was sent for genotyping by TransnetYX: Outsourced PCR Genotyping Services, Cordova, Tennessee, United States of America. Breeding pairs were retired after they had given birth to six litters. Eighteen breeding pairs (8 fed the 30 ppm zinc diet; 10 fed the 150 ppm zinc diet) were utilized to obtain the 93 animals for the behavioural and electrophysiology experiments described in this study (49 wildtypes: 30 males, 19 females; 44 *Shank3*^*−/−*^: 25 males, 19 females). Experiments were conducted on juvenile (3-week-old) and adult (9–10 weeks old for electrophysiology experiments, 16–17 weeks old for behavioural experiments) wildtype and Shank3-KO mice born from control or supplemented maternal zinc diet (MZD). We refer to the supplementation as MZD as although both parents received the zinc diets, males breeders on the zinc supplemented diet would not contribute to prolonged exposure to dietary zinc in the pups unlike female breeders would throughout gestation and lactation. All offspring were weaned at 3 weeks of age onto 30 ppm control diets so the wildtype and Shank3-KO offspring were not exposed to supplemented (150 ppm) zinc diets post-weaning. Both genders were included in all experimental data sets, and wildtype littermates were the control animals in all data sets. The animals used for electrophysiological analysis were independent of mice that were utilized for behavioural studies. All experiments and analyses were performed with the experimenter blinded to genotype and zinc diet by independent animal coding with a unique identification number at weaning.

### Behavioural tests and analysis

Behavioural testing was performed in an isolated room with The Imaging Source Firewire colour camera (SDR Scientific DFK21AF04) recording from the bird’s eye-view and EthoVision XT Version 12.0 (Noldus) video tracking and motion analysis software. Videos were captured at 30 frames per second and 640 × 480 pixel resolution. Animals were habituated in the test room for at least 1 h prior to behavioural testing. The test arena was thoroughly cleaned with 70% ethanol and 2% acetic acid at the beginning of the tests, between animals, and also at the end of the tests.

#### Three-chamber social interaction test

The three-chamber social interaction test enabled evaluation of two critical and distinguishable aspects of social behaviours – social affiliation/motivation (sociability), as well as a social memory for novelty (social novelty recognition) as shown by previous studies [21, 46, 50]. The test was conducted under red light conditions (15 lx) and consisted of three phases as previously described [21, 40, 50, 68]. In phase one, the experimental mouse was placed in the centre chamber, the doors between the three chambers were opened and the mouse could freely explore the entire test arena for 10 min to habituate to the test environment. At the end of the first phase, the mouse was guided back to the centre chamber and the doors closed. For the second phase, which examined the experimental mouse’s sociability, a sex and age-matched stranger C57BL/6 J mouse was placed under either one of the cups located in the side chambers, and once the doors between the chambers were opened, behaviour was recorded for 10 min. The time spent in close interaction was defined as the time the experimental mouse spent sniffing or interacting with the stranger mouse or empty cup. At the end of this sociability phase, the experimental mouse was guided to the centre chamber, the doors closed, and another age and sex-matched stranger mouse was placed under the second empty cup. Once the doors were opened, the experimental mouse’s ability to recognise social novelty was examined in terms of its preference to interact with the novel stranger or the familiar stranger mouse within a 10 min period. Again, time spent in close interaction was defined as the time the experimental mouse spent sniffing or interacting with the novel or familiar stranger mouse. The stranger mice were rested in their home cages for 10 min between tests with water and food, and they were also habituated in the cups for two consecutive days prior to the social interaction test.

#### Grooming behaviour

Animals were placed in a cylindrical arena in a dark room under red light conditions (15 lx) after the animals were habituated in the behavioural room for a minimum of 1 h prior to the test. The mice were then habituated in the test arena for 10 min and their behaviour recorded for 30 min. From the 30-min recording, time spent grooming was analysed. Grooming was defined as wiping, scratching, rubbing or licking of the hands, face, head, ears, body, and tail [15, 21, 49].

#### Dark-light emergence test

The dark-light emergence test was used to assess the anxiety phenotype [21, 36, 45, 50, 59, 65, 73]. This was chosen over the elevated zero maze because mice exhibited genotype- and diet-independent spontaneous jumping behaviour from 2 to 4 weeks of age, also known as the “popcorn” stage [11, 64] – a behaviour that could consequently confound the assessment of anxiety behaviour by three-week-old mice jumping off the elevated zero maze. Tests were conducted under bright white light conditions using a two-chambered apparatus containing a dark chamber (001 lx) and a bright arena (275 lx). The experimental animals were habituated in the dark chamber of the apparatus for 5 min. The door separating the dark and bright chamber was then removed and the mouse could freely move between the two chambers for 10 min. The latency to first enter the bright arena and total time spent in the bright arena were measured and used as indices of anxiety-like behaviours.

### Electrophysiology

#### Acute slice preparation

Acute coronal cortico-striatal brain slices were prepared as described in previous studies [21, 50, 62]. Mice were euthanized with CO_2_ and decapitation, then the brain was removed and submerged in carbogenated (95% oxygen, 5% carbon dioxide) ice-cold protective cutting artificial cerebrospinal fluid (aCSF. in mM: 93 NMDG, 2.5 KCl, 1.2 NaH_2_PO_4_, 30 NaHCO_3_, 20 HEPES, 25 glucose, 2 thiourea, 5 L-ascorbic acid, 3 Na Pyruvate, 0.5 CaCl_2_, 10 MgSO_4_.7H_2_O, pH 7.4, osmolarity 295–305 mOsm). Acute 300 μm coronal slices (Leica VT1200S, blade vibration 1 mm in the x-plane, 0.05 mm/min in the y-plane) were incubated in carbogenated cutting aCSF at 34 °C for 10–12 min to recover. Slices were then maintained at room temperature in carbogenated recovery aCSF (in mM: 97 NaCl, 2.5 KCl, 1.2 NaH_2_PO_4_, 30 NaHCO_3_, 25 glucose, 20 HEPES, 2 CaCl_2_, 2 MgSO_4_.7H_2_O, 2 thiourea, 5 L-ascorbic acid, 3 Na-pyruvate, pH 7.4, osmolarity 295–305 mOsm) for a minimum of 1 h before recording began. Slices were used for a maximum of 8 h and any damaged, discoloured, or unhealthy slices were discarded.

#### Whole-cell patch-clamp recordings

Cortico-striatal slices were transferred into the electrophysiology recording chamber, stabilised using a harp-shaped slice holder, and were perfused using gravity-flow at 2–3 ml/minute with aCSF (in mM: 119 NaCl, 2.5 KCl, 1 Na_2_HPO_4_, 1.3 MgSO_4_, 26.2 NaHCO_3_, 11 D-(+)-glucose, 2.5 CaCl_2_). To stimulate the glutamatergic cortico-striatal pathway into the dorsolateral striatum, a platinum iridium concentric bipolar stimulating electrode was lowered onto the inner border of the corpus callosum between the cortex and the dorsolateral area of the striatum and positioned 3–5 cell body layers deep into the slice. Stimulation was performed with a Digitimer Constant Current Isolated Stimulator (Model DS3; duration 400 μs, frequency 0.1 Hz). Individual medium spiny neurons (MSNs) in the dorsolateral striatum were visualised using a Zeiss Axioskop microscope equipped with infrared differential interference contrast optics and a 40x water immersion objective lens. MSNs were identified by location, shape, and size (ovoid cell body with major axis of 10 to 14 μm). Whole-cell patch-clamp recordings were made with standard wall Borosilicate tubing filamented glass electrodes (Sutter Instrument Company BF150–86-7.5) pulled to 6–8 MΩ resistance using a vertical electrode puller (Narishige PC-10), and filled with internal solution (in mM: 120 K gluconate for AMPAR-mediated EPSCs, or 120 Cs gluconate for NMDAR-mediated EPSCs, 40 HEPES, 5 MgCl_2_, 2 NaATP, 0.3 NaGTP and 5 QX314, pH 7.2, 298 mOsm). The distance between the stimulating electrode and recording electrode was 200–300 μm. Electrophysiological recordings were acquired using a Multiclamp 700B commander (Axon Instruments, California, United States of America) digitized at 10 kHz (Digidata 1440B; Axon Instruments, California, United States of America). Recordings were sampled at 10 kHz and low pass filtered at 1 kHz. Data were acquired and analysed using pClamp 10 acquisition software (Axon Instruments, California, United States of America). The series-resistance was < 20 MΩ. All recordings were performed at room temperature.

AMPAR-mediated EPSCs were measured at − 70 mV at a stimulation intensity that resulted in 50% maximum amplitude [21]. AMPAR-mediated currents were measured for 60 sweeps at 0.1 Hz. The peak amplitudes of monosynaptic currents were measured after the baseline was normalised to zero pA, and averaged across the 60 sweeps to acquire the AMPAR-mediated EPSC amplitude for each neuron. For paired-pulse analysis, two stimuli were separated by 50 ms. PPR was analysed as a ratio of the amplitude of the second response over the first.

MSN NMDAR-mediated EPSCs were recorded at + 40 mV in aCSF + 10 μM CNQX. Presynaptic stimulation was applied at 50 μA pulses applied at 0.1 Hz stimulation. NMDAR-mediated EPSCs were recorded for 30 sweeps. To analyse the peak NMDAR-mediated EPSC amplitude, the baseline was normalised to 0 pA, and the peak current amplitude was measured as a 10 ms window at the peak of the transient, and averaged across the 30 sweeps. Decay kinetics of the NMDAR-mediated responses were measured as previously described [13]. The traces were normalised (Clampfit) and a standard double exponential function was fitted from the peak current to baseline, in which fast current (If) and slow current (Is) are the amplitudes of the fast and slow decay components, and fast time (tf) and slow time (ts) are their respective decay time constants. To compare decay times between different experiments we used a weighted mean decay time constant: tw = [If/(If + Is)]/tf + [Is/(If + Is)]/ts.

### Inductively coupled plasma mass spectrometry (ICP-MS)

Zinc levels were measured in the dietary pellets and the brain samples of the *Shank3*^*+/−*^ mothers as well as their wildtype and *Shank3*^*−/−*^ offspring. All ICP-MS experiments were conducted at the University of Auckland Mass Spectrometry Hub (MaSH) at the School of Chemical Sciences.

#### Tissue collection

Brain samples from *Shank3*^*+/−*^ mothers on the control (30 ppm) or supplemented (150 ppm) zinc diets were collected at the end of their life as breeders (between 39 and 50 weeks of age). Brain samples from wildtype and *Shank3*^*−/−*^ offspring born from these mothers were collected at the end of the behavioural tests (at 9 weeks and 16 weeks old). No 3 week old brain samples were collected as those animals continued onto later time points for the behavioural testing. One hemisphere was flash-frozen using finely-crushed dry ice and subsequently stored at − 80 °C until the ICP-MS experiments were conducted.

#### Sample preparation and procedures

Samples were weighed into 80 mL Teflon tubes and 7 mL of 69% Tracepur HNO3 (Merck) was added. The vessels were sealed and placed in a Maxi-44 rotor and digested in a Ethos-Up Microwave reaction system (Milestone SRL, Italy) at 180C for 20 min. The digest was diluted with 43 mL Type-1 water and a final weight was obtained. The solutions were quantitatively analysed for zinc on an Agilent 7700 ICP-MS in He mode to reduce polyatomic interferences. Calibration standards were prepared in a 10% HNO3 solution from 1000 ppm Single element standards (Inorganic Ventures, USA). A 20 ppb Y solution was used as an online internal standard to monitor for instrument drift and correct for matrix interferences. All results are in μg/g and are back-calculated to the original sample.

### Statistical analysis

Statistical significance was determined using GraphPad Prism Version 7.03. Data were examined for statistical outliers using the Robust regression and Outlier removal (ROUT) method with a Q coefficient of 1%, identifying outliers with a False Discovery Rate less than 1%. Data were also assessed for Gaussian distribution using the Sharpiro-Wilk Normality test, following by two-way analysis of variance (ANOVA) with post hoc multiple comparisons. For two group comparisons, the Student’s t-tests was performed to examine the statistical significance between independent groups. Significant results are marked with * = *p* < 0.05, ** = *p* < 0.01, *** = *p* < 0.001, **** = *p* < 0.0001. All data are presented as means ± the standard error of the mean, and *N* = number of neurons/animals analysed. The specific statistical test details for each data set are provided in the figure legends.

## Results

To first determine whether changes in zinc levels in the maternal diet influenced the development and reproductive competency of *Shank*^*+/−*^ breeders compared to breeders fed control zinc diet, growth was tracked weekly. No significant difference in growth was observed between breeders that were fed with the two different zinc diets (Supplementary Figure 1A). The number of pups born per litter did not significantly vary between breeders fed the control zinc diet (4.21 ± 0.42 pups, *N* = 24 litters) as compared to breeders fed with the supplemented zinc diet (4.88 ± 0.47 pups, *N* = 24 litters, *p*-value = 0.29, Supplementary Figure 1B). With regards to the growth of *Shank3*-wildtype and *Shank3*^*−/−*^ offspring from the maternal supplemented and control zinc diets, we found no significant differences in weight were observed at either 3, 9, or 16 weeks of age (3 weeks: *p*-value = 0.21, Supplementary Figure 1C; 9 weeks: *p*-value = 0.79, Supplementary Figure 1D; 16 weeks: *p*-value = 0.55, Supplementary Figure 1E).

### ASD-related social interaction deficits can be prevented in offspring mice by maternal dietary zinc supplementation in *Shank3*^*−/−*^ mice

To examine the potential of maternal zinc supplementation in preventing the development of ASD-associated deficits in social interaction, the three-chamber social interaction test was utilized to measure sociability and social novelty recognition in juvenile (3 weeks) and adult (16-weeks) *Shank3*^*−/−*^ mice.

**Fig. 1:**
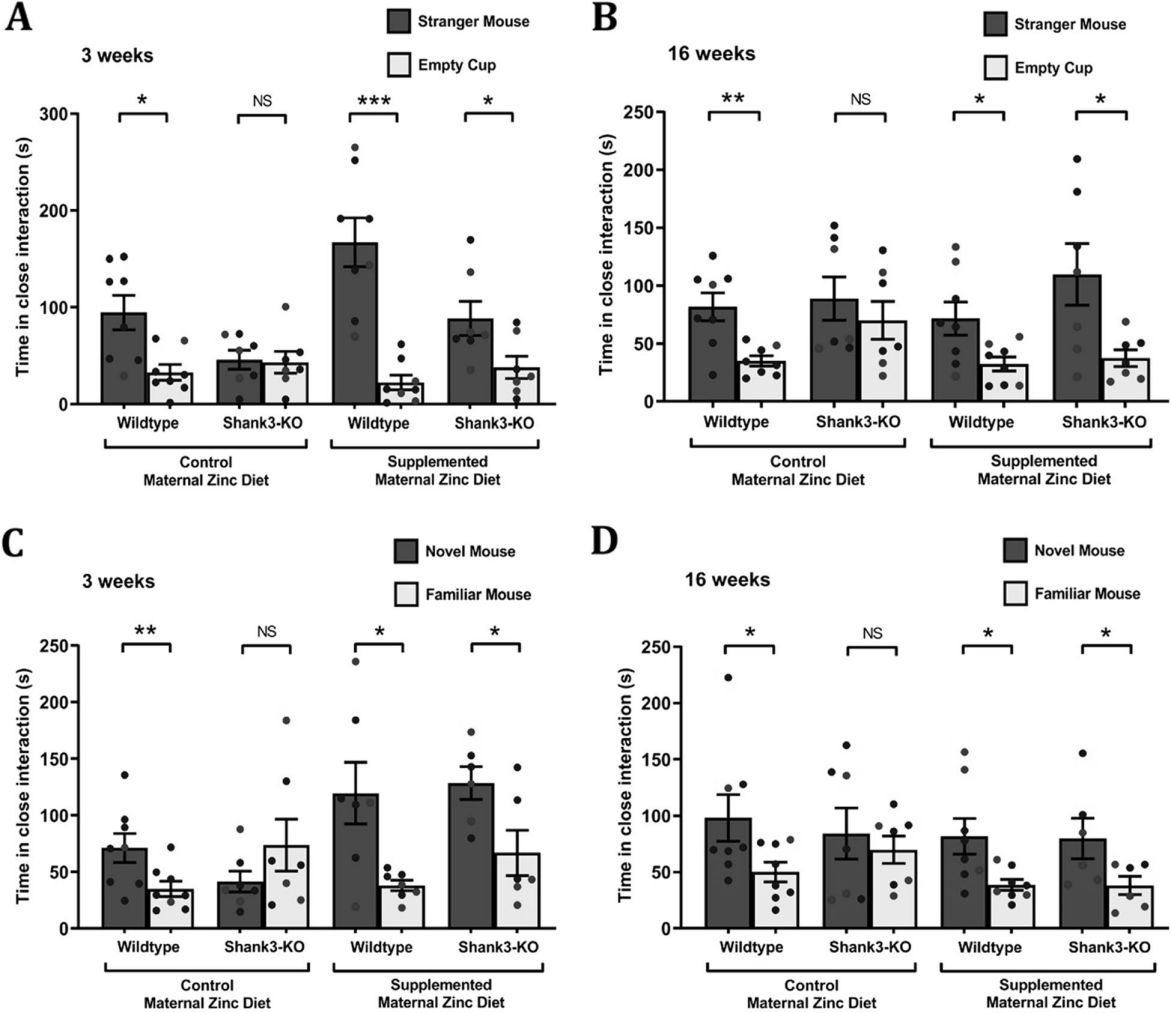
Maternal zinc supplementation prevents deficits in sociability and social novelty recognition in *Shank3*^*−/−*^ offspring mice. Three chamber social interaction test performed in wildtype and *Shank3*^*−/−*^ offspring mice born from mothers on control and supplemented zinc diet. **a** At 3 weeks of age, and **b** at 16 weeks of age, wildtype mice from both maternal zinc diet groups show significantly increased interaction with the stranger mice (*n* = 8 in each group). *Shank3*^*−/−*^ offspring mice born from mothers on the normal zinc diet show no preference for the stranger mice (*n* = 7). Maternal zinc supplementation results in *Shank3*^*−/−*^ mice displaying normal sociability behaviour (*n* = 7). **c** At 3 weeks and **d** at 16 weeks, wildtype mice from both maternal zinc diet groups spend significantly more time in close interaction with the novel mouse than the familiar mouse (*n* = 8 in each group). *Shank3*^*−/−*^ offspring mice born from mothers on the normal zinc diet show no preference for the novel mice (*n* = 7). Maternal zinc supplementation results in *Shank3*^*−/−*^ offspring mice displaying normal social novelty behaviour (*n* = 7). All data represent mean ± standard error of the mean, analysed using two-tailed paired *t*-tests. Each point represents individual mice in each experimental group. NS = not significant, *p < 0.05, **p < 0.01, *** *p* < 0.001

As expected, wildtype offspring mice from the control and supplemented MZD group displayed a significantly greater preference for the stranger mouse than the empty cup at 3 weeks of age. In contrast, *Shank3*^*−/−*^ offspring mice from the control MZD group failed to show this preference, evident of a lack of social behaviour in the mutant mice (Fig. 1a). However, *Shank3*^*−/−*^ offspring mice from the supplemented MZD group did display a significantly greater preference for the stranger mouse than the empty cup (Fig. 1a: wildtype offspring mice from control MZD = stranger mouse: 94.66 ± 17.76 s; empty cup: 32.84 ± 8.15 s, *p*-value = 0.033; *Shank3*^*−/−*^ offspring mice from control MZD = stranger mouse: 45.94 ± 9.76 s, empty cup: 43.28 ± 11.21 s, *p*-value = 0.76; wildtype offspring mice from supplemented MZD = stranger mice: 167.20 ± 25.15; empty cup: 22.43 ± 7.59 s, *p*-value = 0.0005; *Shank3*^*−/−*^ offspring mice from supplemented MZD = stranger mice: 88.64 ± 17.69 s, empty cup: 38.13 ± 11.52 s, *p*-value = 0.033). To determine whether this maternal zinc supplementation-induced prevention of sociability deficits in *Shank3*^*−/−*^ offspring mice could be maintained into adulthood, sociability was again assessed at 16 weeks of age. As expected, wildtype offspring mice from the control and supplemented MZD groups continued to display a significantly greater preference for the stranger mouse than the empty cup, whereas *Shank3*^*−/−*^ offspring mice from the control MZD group exhibited no preference for the stranger mouse versus the empty cup. However, *Shank3*^*−/−*^ offspring mice from the supplemented MZD group demonstrated a significantly greater preference for the stranger mouse than for the empty cup (Fig. 1b: wildtype offspring mice from control MZD = stranger mouse: 81.71 ± 12.03 s; empty cup: 34.99 ± 4.43 s, *p*-value = 0.0026; *Shank3*^*−/−*^ offspring mice from control MZD = stranger mouse: 88.62 ± 18.77 s, 70.03 ± 16.37 s, *p*-value = 0.46; wildtype offspring mice from supplemented MZD = stranger mice: 71.57 ± 14.27; empty cup: 32.25 ± 6.10 s, *p*-value = 0.024; *Shank3*^*−/−*^ offspring mice from supplemented MZD = stranger mice: 109.60 ± 26.55 s, empty cup: 37.44 ± 7.24 s, *p*-value = 0.022).

When social novelty recognition was assessed at 3 weeks of age by introducing a novel mouse to the three-chamber test, wildtype mice born from mothers that were fed with the control and supplemented zinc diet displayed a significantly greater preference for the novel mouse than the familiar mouse (Fig. 1c). In contrast, *Shank3*^*−/−*^ mice from the control MZD exhibited no preference between the novel mouse and the familiar mouse, demonstrating the expected social novelty recognition deficit previously observed [21, 50]. However, *Shank3*^*−/−*^ offspring mice from the supplemented MZD group demonstrated a significantly greater preference for the novel mouse than for the familiar mouse (Fig. 1c: wildtype offspring mice from control MZD = novel mouse: 71.13 ± 12.83 s; familiar mouse: 35.00 ± 6.73 s, *p*-value = 0.0092; *Shank3*^*−/−*^ offspring mice from control MZD = novel mouse: 41.41 ± 9.21 s, familiar mouse: 73.65 ± 22.95 s, *p*-value = 0.28; wildtype offspring mice from supplemented MZD = novel mice: 119.60 ± 27.25 s; familiar mouse: 38.02 ± 4.60 s, *p*-value = 0.018; *Shank3*^*−/−*^ offspring mice from supplemented MZD = novel mice: 128.60 ± 14.50 s, familiar mice: 66.74 ± 20.02 s, *p*-value = 0.034). When tested again in adult mice at 16 weeks of age, wildtype mice born from mothers that were fed with the control and supplemented zinc diet displayed a significantly greater preference for the novel mouse than the familiar mouse, while *Shank3*^*−/−*^ mice born from the control MZD exhibited no preference between the novel mouse and the familiar mouse. However, again *Shank3*^*−/−*^ mice from the supplemented MZD group demonstrated a significantly greater preference for the novel mouse than for the familiar mouse (Fig. 1d: wildtype offspring mice from control MZD = novel mouse: 98.08 ± 20.80 s; familiar mouse: 50.01 ± 8.77 s, *p*-value = 0.05; *Shank3*^*−/−*^ offspring mice from control MZD = novel mouse: 84.17 ± 22.72 s, familiar mouse: 69.83 ± 12.18 s, *p*-value = 0.63; wildtype offspring mice from supplemented MZD = novel mice: 81.70 ± 15.90 s; familiar mouse: 38.55 ± 4.87 s, *p*-value = 0.023; *Shank3*^*−/−*^ offspring mice from supplemented MZD = novel mice: 79.78 ± 18.06 s, 38.04 ± 8.14 s, *p*-value = 0.039), thereby demonstrating that the rescuing effect on social behaviour induced by maternal zinc supplementation can be maintained into adulthood.

### ASD-related excessive grooming behaviours can be reversed by maternal dietary zinc supplementation in the adult *Shank3*^*−/−*^ offspring mice

**Fig. 2:**
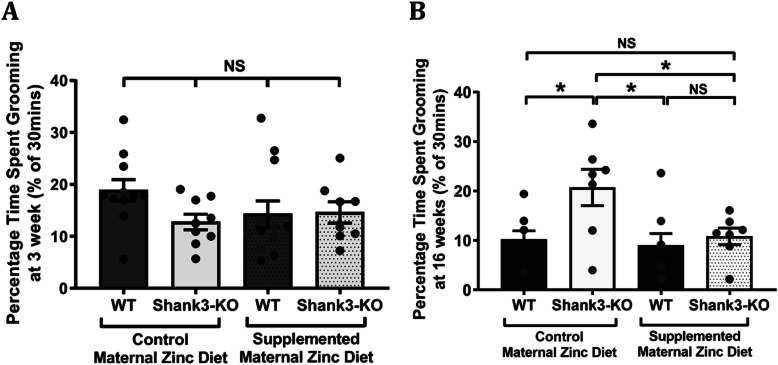
Maternal zinc supplementation prevents repetitive grooming behaviour in adult offspring *Shank3*^*−/−*^ mice. Repetitive grooming behaviours, as measured by the percentage of time spent wiping, scratching, rubbing or licking of the hands, face, head, ears, body or tail, measured in wildtype and *Shank3*^*−/−*^ offspring mice from mothers fed normal and supplemented zinc levels. **a** No significant difference was observed in wildtype (WT) and *Shank3*^*−/−*^ mice at 3 weeks of age (WT control maternal zinc diet (MZD) *n* = 11, *Shank3*^*−/−*^ control MZD *n* = 9, WT supplemented MZD *n* = 12, *Shank3*^*−/−*^ supplemented MZD *n* = 8). **b** At 16 weeks of age increased repetitive grooming behaviour was observed in *Shank3*^*−/−*^ offspring mice from the control maternal zinc diet group (WT *n* = 8, *Shank3*^*−/−*^*n* = 7), and this was no longer present in *Shank3*^*−/−*^ offspring mice in the supplemented maternal zinc diet group (WT *n* = 8, *Shank3*^*−/−*^*n* = 7). All data represent mean ± standard error of the mean, analysed using two-way ANOVA with Tukey’s multiple comparisons test. All dots represent data points from individual mice. NS = not significant, *p < 0.05

At the juvenile stage, there was no significant difference in the percentage of time spent grooming between mice born from mothers that were fed with the control zinc diet (Fig. 2a: wildtype: 18.88 ± 2.06%,; *Shank3*^*−/−*^: 12.80 ± 1.49%) or mice born from mothers that were fed with the supplemented zinc diet (wildtype: 14.29 ± 2.54%; *Shank3*^*−/−*^: 14.62 ± 2.05%, *p*-value = 0.23), showing that the repetitive behaviour phenotype is not present in juvenile *Shank3*^*−/−*^ mice. In adult offspring mice, as expected *Shank3*^*−/−*^ mice from mothers that were fed with the control zinc diet spent a significantly greater time grooming (20.71 ± 3.69%) in comparison to wildtype mice from mothers that were fed with the control zinc diet (10.21 ± 1.72%, *p*-value = 0.041, Fig. 2b). Interestingly, maternal zinc supplementation prevented excessive grooming behaviours in adult *Shank3*^*−/−*^ offspring mice as there was no significant difference in the percentage time spent grooming between wildtype (9.00 ± 2.42%) and *Shank3*^*−/−*^ offspring mice (10.81 ± 1.67%, *p*-value = 0.95) from mothers that were fed with the supplemented zinc diet.

### Maternal zinc supplementation prevents anxiety-associated behaviours in *Shank3*^*−/−*^ offspring mice

**Fig. 3:**
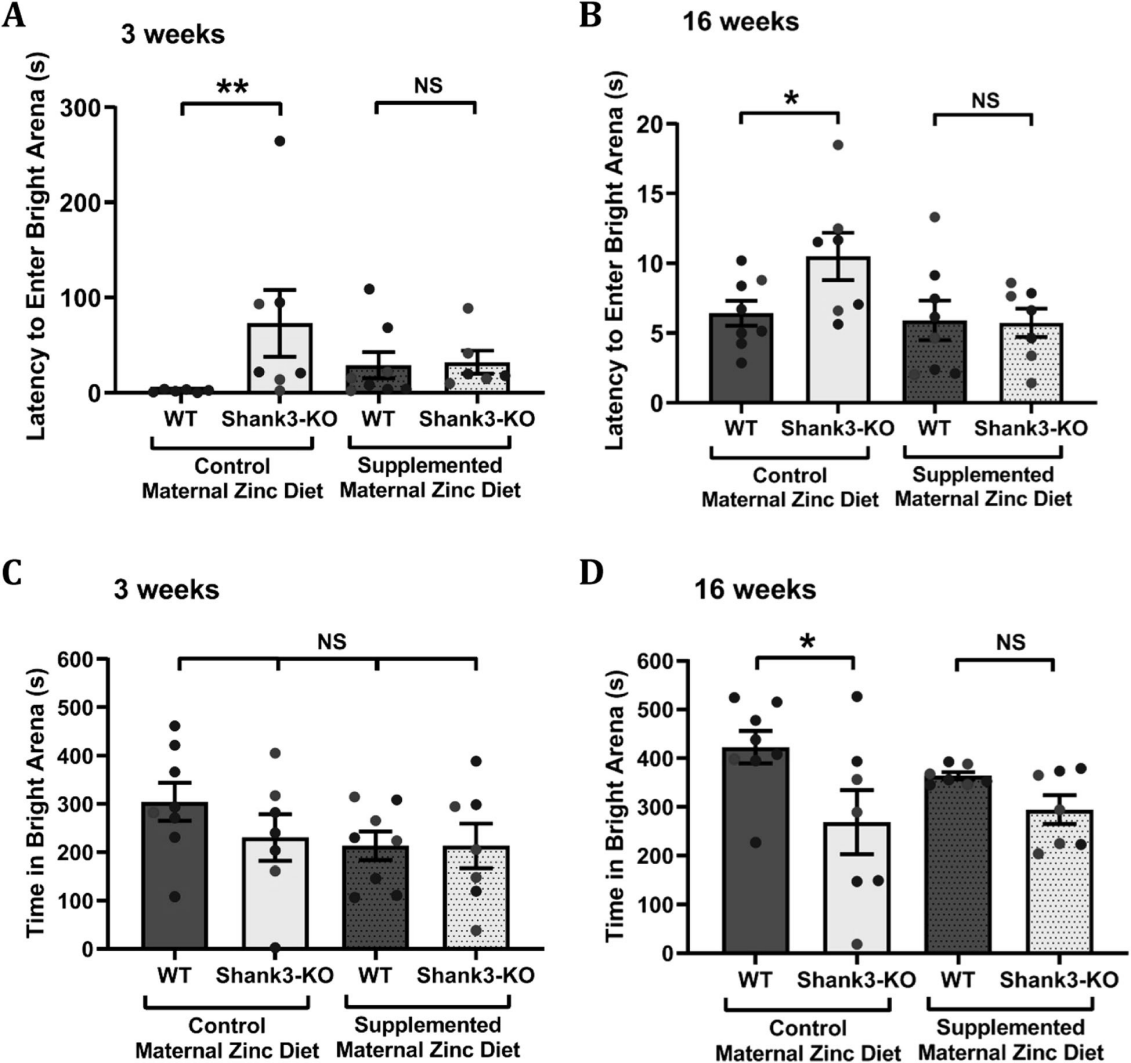
Influence of maternal zinc supplementation on anxiety of *Shank3*^*−/−*^ offspring mice. Dark-light emergence test measured in wildtype (WT) and *Shank3*^*−/−*^ offspring mice. **a** At 3 weeks and **b** at 16 weeks of age, *Shank3*^*−/−*^ offspring mice took a significantly longer time to enter the bright arena in comparison to WT from the control maternal zinc diet. No statistical difference in latency to enter the bright arena was observed between WT and *Shank3*^*−/−*^ offspring mice from the supplemented maternal zinc diet (3 weeks: WT control maternal zinc diet (MZD) *n* = 6, *Shank3*^*−/−*^ control MZD *n* = 7, WT supplemented MZD *n* = 8, *Shank3*^*−/−*^ supplemented MZD *n* = 6; 16 weeks: WT MZD *n* = 8, *Shank3*^*−/−*^ control MZD *n* = 7, WT supplemented MZD *n* = 8, *Shank3*^*−/−*^ supplemented MZD *n* = 7). **c** At 3 weeks, there is no difference in time spent in the bright arena regardless of genotype or maternal zinc diet (WT control MZD *n* = 8, *Shank3*^*−/−*^ control MZD *n* = 7, WT supplemented MZD *n* = 8, *Shank3*^*−/−*^ supplemented MZD *n* = 7). **d** At 16 weeks of age, *Shank3*^*−/−*^ offspring mice from the control maternal zinc diet spent a significantly reduced amount of time in the bright arena compared to wildtype mice. However, no significant difference was observed in time spent in the bright arena between WT and *Shank3*^*−/−*^ mice from the supplemented maternal zinc diet group (WT control MZD *n* = 8, *Shank3*^*−/−*^ control MZD *n* = 7, WT supplemented MZD *n* = 7, *Shank3*^*−/−*^ supplemented MZD *n* = 7). All data represent mean ± standard error of the mean, analysed using two-way ANOVA with Tukey’s multiple comparisons test. All data points represent individual mice. NS = not significant, *p < 0.05, **p < 0.01

The dark-light emergence test was used to examine anxiety-associated behaviours in wildtype and *Shank3*^*−/−*^ mice. In juvenile mice, *Shank3*^*−/−*^ mice born from mothers that were fed with the control zinc diet took significantly longer to exit the dark arena and enter the bright arena (Fig. 3a; 73.10 ± 35.00 s) in comparison to wildtype from mothers that were fed with the control zinc diet (Fig. 3a; 2.05 ± 0.62 s, *p*-value = 0.0098). In mice born from mothers that were fed the supplemented zinc diet however, there was no significant difference in the latency to enter the bright arena between wildtype (29.14 ± 13.75 s) and *Shank3*^*−/−*^ (32.18 ± 12.18 s, *p*-value > 0.99) mice, showing a prevention of this anxiety behaviour. Similarly, adult *Shank3*^*−/−*^ mice from mothers that were fed with the control zinc diet took significantly longer to enter the bright arena (10.50 ± 1.70 s) than wildtype mice from mothers that were fed with the control zinc diet (6.42 ± 0.89 s, *p*-value = 0.046; Fig. 3b). However, there was no significant difference in the latency to enter the bright arena between wildtype (5.91 ± 1.41 s) and *Shank3*^*−/−*^ mice (5.74 ± 1.01 s, *p*-value = 0.99) from mothers that were fed with the supplemented zinc diet.

We also measured the total time spent in the bright arena. At 3 weeks of age (Fig. 3c), no significant difference was observed between wildtype or *Shank3*^*−/−*^ mice from either mothers that were fed with the control zinc diet (wildtype: 304.70 ± 39.63 s; *Shank3*^*−/−*^: 230.60 ± 48.4 s), or from mothers that were fed with the supplemented zinc diet (wildtype: 213.50 ± 29.53 s; *Shank3*^*−/−*^: 213.50 ± 45.88 s, *p*-value = 0.33). Adult mutant mice did show a significant phenotype, with *Shank3*^*−/−*^ mice from mothers that were fed with the control zinc diet spending a significantly reduced amount of time in the bright arena (268.80 ± 65.79 s) in comparison to wildtype mice from mothers that were fed with the control zinc diet (432.00 ± 33.29 s, *p*-value = 0.045; Fig. 3d). However, in the maternal zinc supplemented groups, there was no significant difference in time spent in the bright arena between wildtype mice from supplemented MZD (364.30 ± 7.35) and *Shank3*^*−/−*^ mice from supplemented MZD (294.90 ± 29.54 s, *p*-value = 0.62).

### Maternal dietary zinc supplementation alters synaptic function in *Shank3*^*−/−*^ adult offspring mice

**Fig. 4:**
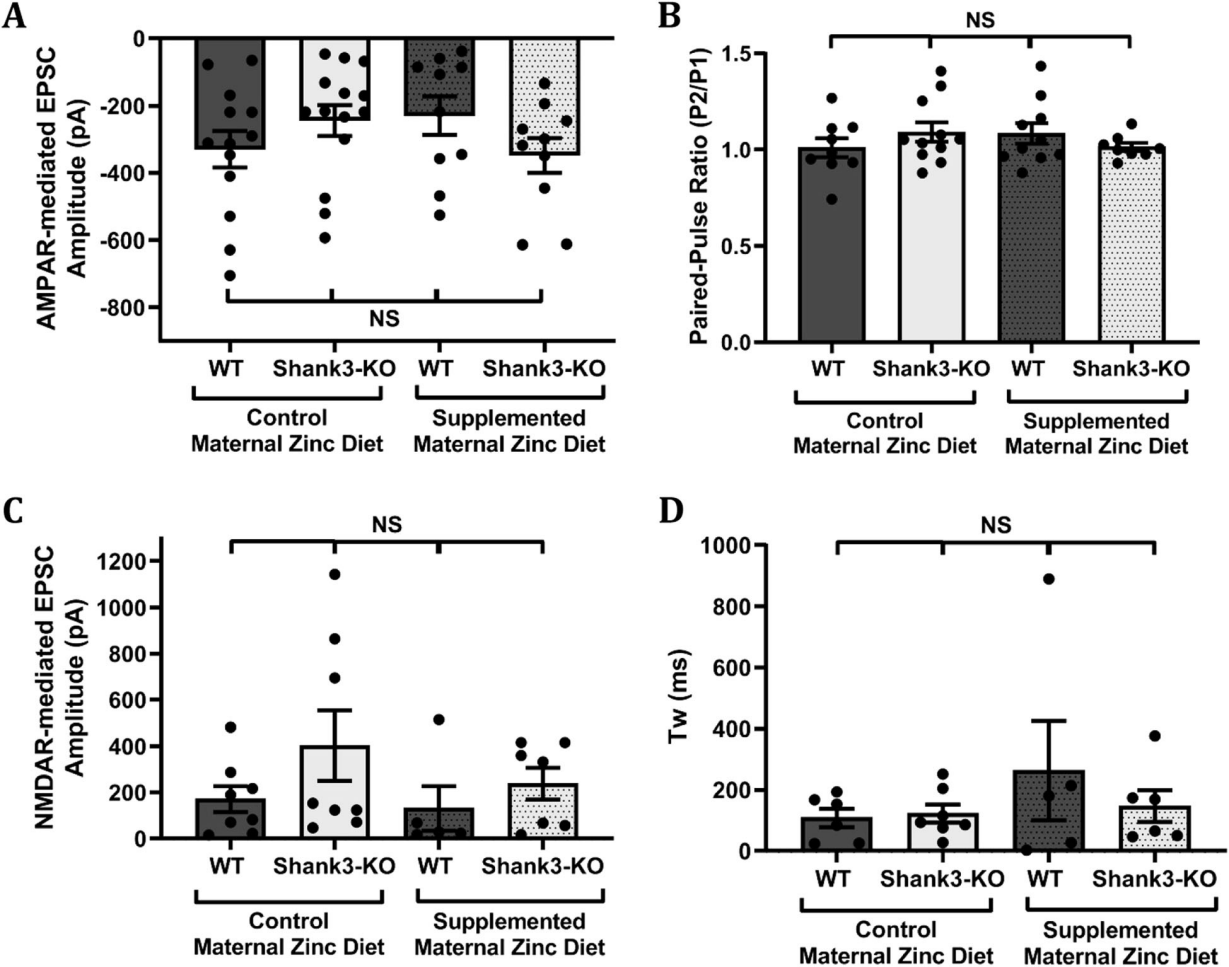
Maternal zinc supplementation does not alter cortico-striatal glutamatergic synapse function in three-week-old *Shank3*^*−/−*^ offspring mice. MSN striatal evoked AMPAR- and NMDAR-mediated EPSC amplitudes measured in 3 week old wildtype (WT) and *Shank3*^*−/−*^ offspring mice from normal and supplemented maternal zinc diet groups. **a** No significant differences were observed in AMPAR EPSC amplitudes between WT or *Shank3*^*−/−*^ offspring mice from either maternal zinc diet group (WT control maternal zinc diet (MZD) *n* = 13 neurons from 3 mice, *Shank3*^*−/−*^ control MZD *n* = 14 neurons from 4 mice, WT supplemented MZD *n* = 10 neurons from 2 mice, *Shank3*^*−/−*^ supplemented MZD *n* = 10 neurons from 2 mice). **b** No significant differences were observed in paired-pulse ratio between either genotypes or maternal dietary zinc groups (WT control MZD *n* = 9 neurons from 3 mice, *Shank3*^*−/−*^ control MZD *n* = 11 neurons from 4 mice, WT supplemented MZD *n* = 10 neurons from 2 mice, *Shank3*^*−/−*^ supplemented MZD *n* = 8 neurons from 2 mice). **c** Evoked NMDAR-mediated EPSC amplitudes in WT and *Shank3*^*−/−*^ offspring mice from either maternal zinc group were not significantly different (WT control MZD *n* = 8 neurons from 3 mice, *Shank3*^*−/−*^ control MZD *n* = 8 neurons from 4 mice, WT supplemented MZD *n* = 5 neurons from 2 mice, *Shank3*^*−/−*^ supplemented MZD *n* = 7 neurons from 2 mice). **d** Decay kinetics, measured as the weighted time constant tau (t_w_), were also not significantly different across experimental groups (WT control MZD *n* = 6 neurons from 3 mice, *Shank3*^*−/−*^ control MZD *n* = 7 neurons from 4 mice, WT supplemented MZD *n* = 5 neurons from 2 mice, *Shank3*^*−/−*^ supplemented MZD *n* = 7 neurons from 2 mice). All values are presented as mean ± standard error of the mean. Individual data points represent individual neurons, and were statistically analysed using two-way ANOVA with Tukey’s multiple comparisons test. NS = not significant

In juvenile mice, no significant difference was observed in evoked AMPAR-mediated EPSC amplitudes between wildtype and *Shank3*^*−/−*^ mice from mothers on the control or supplemented zinc diet (Fig. 4a, average EPSC amplitudes were wildtype from control MZD: − 329.70 ± 54.48 pA; *Shank3*^*−/−*^ mice from control MZD: − 244.00 ± 46.11 pA; wildtype from supplemented MZD: − 229.40 ± 57.41 pA; *Shank3*^*−/−*^ mice from supplemented MZD: − 348.20 ± 51.58 pA; *p*-value = 0.31). Maternal dietary zinc supplementation also did not alter the paired-pulse ration (PPR) in juvenile wildtype and *Shank3*^*−/−*^ mice (Fig. 4b, average PPRs were: wildtype from control MZD 1.01 ± 0.05,; *Shank3*^*−/−*^ mice from control MZD 1.09 ± 0.05 pA; wildtype from supplemented MZD 1.09 ± 0.05 pA; *Shank3*^*−/−*^ mice from supplemented MZD 1.01 ± 0.02 pA; *p*-value = 0.48). No significant differences in evoked NMDAR-mediated EPSC amplitude and decay kinetics were evident between wildtype and *Shank3*^*−/−*^ mice from mothers that were fed with the control or supplemented zinc diet (Fig. 4c, d). Average NMDAR-mediated EPSC amplitudes were: wildtype from control MZD 170.60 ± 56.31 pA; *Shank3*^*−/−*^ mice from control MZD 402.70 ± 152.50 pA; wildtype from supplemented MZD 130.50 ± 96.41 pA; *Shank3*^*−/−*^ mice from supplemented MZD 238.00 ± 68.64 pA; *p*-value = 0.28. Average NMDAR-mediated EPSC weighted tau were: wildtype from control MZD: 108.20 ± 30.17 ms; *Shank3*^*−/−*^ mice from control MZD: 122.20 ± 29.68 ms; wildtype from supplemented MZD: 262.90 ± 162.30 ms; *Shank3*^*−/−*^ mice from supplemented MZD: 147.00 ± 51.65 ms; *p*-value = 0.51.

**Fig. 5:**
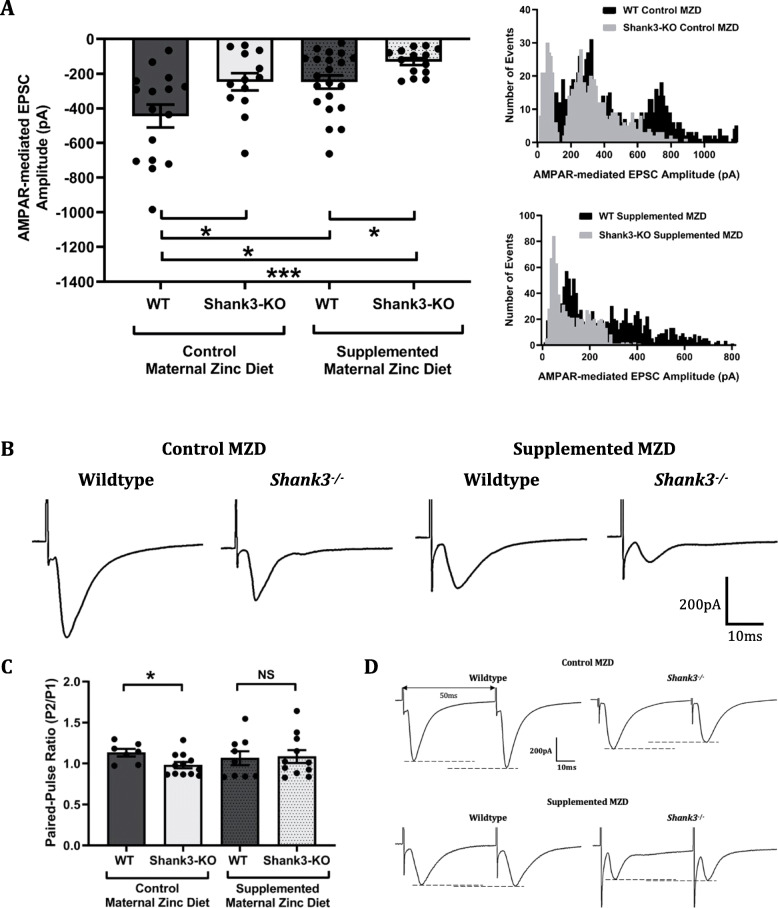
Effect of maternal zinc supplementation on cortico-striatal AMPAR-mediated EPSCs in adult *Shank3*^*−/−*^ offspring mice. MSN striatal evoked AMPAR-mediated EPSC amplitudes measured in adult wildtype (WT) and *Shank3*^*−/−*^ offspring mice from normal and supplemented maternal zinc diet groups. **a** Left: In comparison to WT, adult *Shank3*^*−/−*^ mice from the control or supplemented maternal zinc diet (MZD) group displayed significantly decreased evoked AMPAR-mediated ESPC amplitudes. Right: Frequency histogram of AMPAR-mediated EPSC amplitudes illustrating a shift towards smaller AMPAR-mediated EPSC amplitudes in *Shank3*^*−/−*^ mice from mothers on the control (top) or supplemented (bottom) zinc diet. WT control MZD *n* = 16 neurons from 7 mice, *Shank3*^*−/−*^ control MZD *n* = 13 neurons from 7 mice, WT supplemented MZD *n* = 21 neurons from 10 mice, *Shank3*^*−/−*^ supplemented MZD *n* = 14 neurons from 5 mice. **b** Representative AMPAR-mediated EPSC traces from adult WT and *Shank3*^*−/−*^ mice from mothers that were fed with the control zinc diet (left), and WT and *Shank3*^*−/−*^ mice from mothers that were fed with the supplemented zinc diet (right). **c** Adult *Shank3*^*−/−*^ mice from the control maternal zinc diet (MZD) have a significantly reduced paired-pulse ratio (PPR) in comparison to wildtype (WT) mice. No significant difference was observed in PPR between *Shank3*-wildtype and *Shank3*^*−/−*^ mice from mothers that were fed with the supplemented zinc diet. WT control MZD *n* = 7 neurons from 3 mice, *Shank3*^*−/−*^ control MZD *n* = 12 neurons from 5 mice, WT supplemented MZD *n* = 9 neurons from 3 mice, *Shank3*^*−/−*^ supplemented MZD *n* = 11 neurons from 4 mice. **d** Representative traces of PPR from wildtype (top left) and *Shank3*^*−/−*^ mice (top right) from mothers that were fed with the control zinc diet, and wildtype (bottom left) and *Shank3*^*−/−*^ mice (bottom right) from mothers that were fed with the supplemented zinc diet. All values are presented as mean ± standard error of the mean. Individual data points represent individual neurons, and were statistically analysed using two-way ANOVA with Tukey’s multiple comparisons test. NS = not significant, *p < 0.05, *** *p* < 0.001

In adult mice however, evoked AMPAR-mediated EPSCs were significantly decreased in *Shank3*^*−/−*^ offspring mice in comparison to wildtype offspring mice from mothers that were fed with the control or the supplemented zinc diet (Fig. 5a, b). In addition, AMPAR-mediated EPSCs were also significantly decreased in wildtype and *Shank3*^*−/−*^ offspring from mothers that were fed with the supplemented zinc diet in comparison to wildtype mice born from mothers that were fed with the control zinc diet (average EPSC amplitudes: wildtype mice from control MZD -444.00 ± 65.77 pA; *Shank3*^*−/−*^ mice from control MZD -246.80 ± 49.75 pA; *p*-value = 0.029; wildtype mice from supplemented MZD -248.40 ± 38.55 pA; *Shank3*^*−/−*^ mice from supplemented MZD -130.00 ± 19.82 pA; *p*-value = 0.024). This was also reflected in the left-shifted amplitude-frequency histograms demonstrating a higher frequency of smaller AMPAR-mediated EPSCs from *Shank3*^*−/−*^ mice born from mothers that were fed with the control or supplemented zinc diet. In comparison to wildtype mice from mothers that were fed with the control zinc diet, AMPAR-mediated EPSCs were significantly reduced in wildtype mice from mothers that were fed with the supplemented zinc diet (*p*-value = 0.014), and were also significantly reduced in *Shank3*^*−/−*^ mice from mothers that were fed with the supplemented zinc diet (*p*-value = 0.0001). Adult *Shank3*^*−/−*^ mice from mothers that were fed with the control zinc diet also displayed a reduced PPR in comparison to wildtype mice from mothers on the same control zinc diet (wildtype: 1.132 ± 0.046; *Shank3*^*−/−*^: 0.98 ± 0.039, *p*-value = 0.025, Fig. 5c, d). However, this difference in PPR was not observed between wildtype and *Shank3*^*−/−*^ mice from mothers that were fed with the supplemented zinc diet (wildtype from supplemented MZD: 1.065 ± 0.084; *Shank3*^*−/−*^ mice from supplemented MZD: 1.085 ± 0.078, *p*-value = 0.86).

**Fig. 6:**
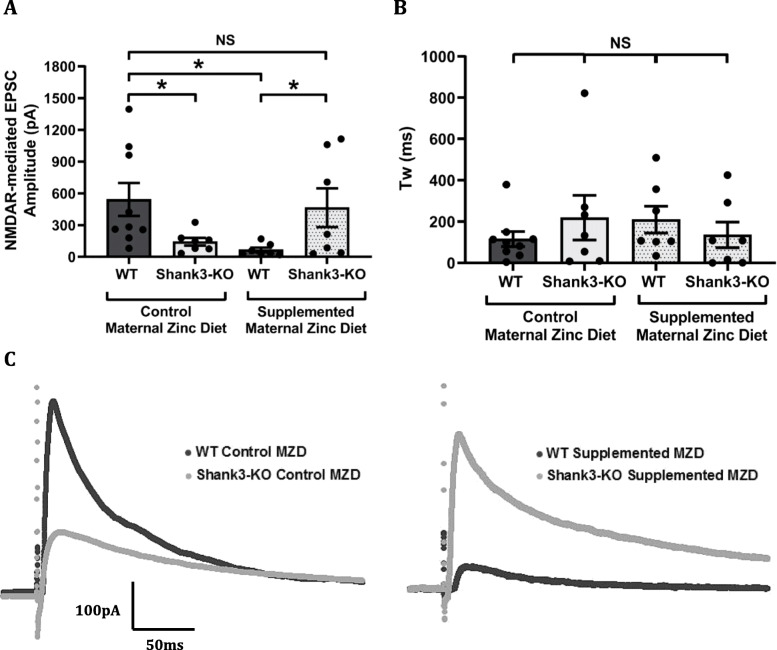
Effect of maternal zinc supplementation on cortico-striatal NMDAR-mediated EPSCs in adult *Shank3*^*−/−*^ mice. Adult MSN NMDAR-mediated EPSC amplitudes measured in adult wildtype (WT) and *Shank3*^*−/−*^ offspring mice from normal and supplemented maternal zinc diet groups. **a** Evoked NMDAR-mediated EPSC amplitudes are significantly reduced in adult *Shank3*^*−/−*^ offspring mice from mothers that were fed with the control zinc diet in comparison to adult wildtype mice from mothers that were fed with the control zinc diet. The supplemented maternal zinc diet also induced a significant decrease in adult WT mice. NMDAR-mediated EPSC amplitudes were comparable between WT mice from mothers that were fed with the control zinc diet and *Shank3*^*−/−*^ mice from mothers that were fed with the supplemented zinc diet. **b** NMDAR decay kinetics, measured as weighted tie constant tau (t_w_), were not significantly different between adult WT and *Shank3*^*−/−*^ mice from mothers that were fed with the control or supplemented zinc diet. **c** Representative NMDAR-mediated EPSC traces from adult WT and *Shank3*^*−/−*^ mice from mothers that were fed with the control zinc diet (left), and WT and *Shank3*^*−/−*^ mice from mothers that were fed with the supplemented zinc diet (right). WT control MZD *n* = 9 neurons from 5 mice, *Shank3*^*−/−*^ control MZD *n* = 7 neurons from 5 mice, WT supplemented MZD *n* = 7 neurons from 3 mice, *Shank3*^*−/−*^ supplemented MZD *n* = 7 neurons from 4 mice. All values are presented as mean ± standard error of the mean. Individual data points represent individual neurons, and were statistically analysed using two-way ANOVA with Tukey’s multiple comparisons test. NS = not significant, *p < 0.05

Evoked NMDAR-mediated EPSC amplitudes were significantly decreased in adult *Shank3*^*−/−*^ mice from mothers that were fed with the control zinc diet (143.10 ± 36.51 pA) in comparison to the wildtype mice from mothers that were fed with the control zinc diet (542.50 ± 155.40 pA, *p*-value = 0.044, Fig. 6a). In contrast, *Shank3*^*−/−*^ mice from mothers that were fed with the supplemented zinc diet had significantly increased NMDAR-mediated EPSC amplitudes (464.20 ± 183.30 pA) in comparison to the wildtype mice from mothers that were fed with the supplemented zinc diet (66.23 ± 21.19 pA, *p*-value = 0.05). However, the wildtype offspring mice from the supplemented maternal zinc diet also displayed significantly reduced NMDAR-mediated EPSC amplitudes in comparison to wildtype mice (*p*-value = 0.034), but *Shank3*^*−/−*^ mice from mothers that were fed with the supplemented zinc diet displayed comparable NMDAR-mediated EPSC amplitudes. The decay kinetics of evoked NMDAR-mediated EPSCs were not significantly different between wildtype or *Shank3*^*−/−*^ mice from mothers that were fed with the control or supplemented zinc diet (Fig. 6b). NMDAR-mediated EPSC weighted tau were: wildtype from control MZD 115.40 ± 36.19 ms; *Shank3*^*−/−*^ mice from control MZD 218.80 ± 107.90 ms; wildtype from supplemented MZD 209.70 ± 64.94 ms; *Shank3*^*−/−*^ mice from supplemented MZD 136.00 ± 61.83 ms; *p*-value = 0.63.

### Brain zinc levels measured using inductively coupled plasma mass spectrometry (ICP-MS)

Surprisingly, no significant difference was detected in the whole-brain zinc levels between the *Shank*^*+/−*^ mothers fed the control zinc diet (18.34 ± 0.8736 μg/g zinc, *N* = 4 brains) or the supplemented zinc diet (17.51 ± 1.349 μg/g zinc, *N* = 3 brains, *p*-value = 0.608). Zinc levels in the brains of 9 week old *Shank3*-wildtype or *Shank3*^*−/−*^offspring mice were also not significantly different regardless of whether they were born from mothers fed the control or the supplemented zinc diet (*Shank3-*wildtype from control MZD: 13.45 ± 1.19 μg/g; *Shank3*^*−/−*^ mice from control MZD: 15.76 ± 0.5864 μg/g; *Shank3-*wildtype from supplemented MZD: 16.12 ± 0.273 μg/g; *Shank3*^*−/−*^ mice from supplemented MZD: 17.70 ± 1.806 μg/g; *N* = 3 brains in each group; *p*-value = 0.141). Finally, zinc levels in the brains of 16 week old *Shank3*-wildtype or *Shank3*^*−/−*^offspring mice were not significantly different regardless of whether they were born from mothers fed the control or the supplemented zinc diet (*Shank3-*wildtype from control MZD: 16.10 ± 0.2656 μg/g; *Shank3*^*−/−*^ mice from control MZD: 17.15 ± 0.8517 μg/g; *Shank3-*wildtype from supplemented MZD: 17.04 ± 0.9199 μg/g; *Shank3*^*−/−*^ mice from supplemented MZD: 17.45 ± 0.5385 μg/g; *N* = 3 brains in each group; *p*-value = 0.5798).

## Discussion

Our research has demonstrated that dietary maternal zinc supplementation from the beginning of brain development has the ability to prevent ASD-associated behavioural deficits at 3 weeks of age with benefits lasting into adulthood. We also observed functional synaptic changes at the glutamatergic cortico-striatal pathway in response to changes in maternal dietary zinc levels, particularly in adult *Shank3*^*−/−*^ offspring mice with changes in postsynaptic NMDA receptor-mediated currents and presynaptic function. These data show that increased maternal dietary zinc during pregnancy and lactation can alter the development of ASD-associated changes at the synaptic and the behavioural levels in both young and adult offspring.

### Maternal zinc supplementation prevents behavioural deficits in social interaction, repetitive grooming behaviours and anxiety in *Shank3*^*−/−*^ offspring mice

ASD-associated deficits in sociability and anxiety were evaluated using the three-chamber social interaction test and the light-dark emergence test. Formerly, ASD-associated behavioural tests in *Shank3*^*−/−*^ mice have been conducted from 5 weeks of age up to 8 months [10, 21, 33, 36, 40, 50, 57, 59, 65, 66, 73]. Here we reveal (1) that anxiety, deficits in sociability, and social novelty recognition are present in *Shank3*^*−/−*^ mice as early as 3 weeks of age, (2) that the development of anxiety, repetitive behaviours, sociability, and social novelty recognition behaviours can not only be prevented if zinc supplementation is initiated from the beginning of brain development, but moreover are sustained into adulthood long after the exposure to zinc supplementation had ceased. This suggests that maternal zinc supplementation caused changes in the developing brain of *Shank3*^*−/−*^ offspring that led to a sustained prevention of ASD-associated repetitive behaviours, anxiety, and deficits in social interaction. These data significantly extend from our previous finding that dietary zinc supplementation performed directly in post-weaned mice prevents deficits in social novelty recognition, excessive grooming, and increased anxiety in adolescent *Shank3*^*−/−*^ mice [21]. Recently, several different approaches have been used to reverse ASD-associated behavioural deficits in *Shank3*^*−/−*^ models of ASD. Two independent studies modulated mGluR5 activity using CDPPB to rescue behavioural deficits observed in *Shank3* knockout mice [63, 65]. Vicidomini et al. [63] observed an improvement in social deficits and excessive grooming in *Shank3*^*∆*ex11B^ mice, whereas Wang et al. [65] observed a partial rescue in instrumental learning in *Shank3*^*∆*ex4-22J^ mice. In *Shank3* knockout rats, oxytocin treatment was able to ameliorate deficits in long-term social memory and attention deficits [29]. The results from these different approaches are highly encouraging. It is also noteworthy that only dietary zinc supplementation has been shown to rescue or prevent all ASD-associated behavioural phenotypes in *Shank3*^*−/−*^ models of ASD.

We did not observe a repetitive grooming phenotype in juvenile *Shank3*^*−/−*^ offspring mice from the control or supplemented MZD groups. A possible reason may be that up until this age experimental mice were unweaned, and the mothers likely played a more dominant role in grooming their offspring as compared to adult *Shank3*^*−/−*^ offspring mice who did display this phenotype [10, 16, 21, 33, 36, 45, 50, 57, 65, 66, 73]. This is likely because repetitive grooming is an ASD phenotype that worsens with age [50], and maternal zinc supplementation also prevented this excessive repetitive grooming in adult *Shank3*^*−/−*^ mice.

### Effects of maternal zinc supplementation on cortico-striatal synaptic physiology

The cortico-striatal glutamatergic pathway has been implicated in repetitive behaviours, and the striatal growth rate has been found to be increased in children with ASD [37, 38, 42]. Zinc supplementation has been shown to alter synapse function in the cortico-striatal pathway: Fourie et al. [21] observed increased synaptic Shank2 expression, decreased NMDAR-mediated EPSC amplitude, and impaired LTP at cortico-striatal synapses in *Shank3*^*−/−*^ mice fed with the zinc supplemented diet, and these zinc-induced synaptic changes in the cortico-striatal pathway may contribute to the prevention of ASD-associated repetitive behaviours.

We have observed that maternal dietary zinc supplementation appears to influence synaptic physiology in a different manner to that observed previously with direct post-weaning dietary zinc supplementation occurring up until 2 months of age [21]. In juvenile 3 week old mice, neither *Shank3* knockout nor maternal zinc supplementation significantly altered AMPAR-mediated EPSCs, NMDAR-mediated EPSCs, or PPR in the cortico-striatal pathway as previously described in post-weaning dietary zinc experiments. These differences likely reflect a potential developmental difference in zinc-dependent regulation of glutamatergic synaptic function (pre-natal, post-natal, versus post-weaning), and/or differences in the level of neuronal zinc exposure due to delivery via direct dietary intake versus via the placenta and lactation.

The lack of change in glutamatergic cortico-striatal synaptic function at 3 weeks of age aligns with the lack of ASD-associated grooming behaviours in these juvenile mice, as this pathway is implicated in repetitive behaviours [37, 38, 42]. However, in adult *Shank3*^*−/−*^ mice born from mothers that were fed with the control zinc diet, both AMPA and NMDA receptor activity was significantly reduced at glutamatergic synapses onto dorsolateral striatal MSNs. These data emphasise that Shank3 plays a critical role at cortico-striatal synapses, and lack of Shank3 significantly decreases the activity of glutamatergic synapses onto MSNs in adult mice. This is likely due to the major scaffolding role Shank3 plays within the postsynaptic density where it interacts with not only glutamatergic receptors, but also forms postsynaptic signalling complexes that are capable of altering both postsynaptic and also presynaptic function [4]. At wildtype synapses, Shank3 has been shown to increase the localisation and stabilisation of synaptic NMDARs by crosslinking NMDAR/PSD95 complexes with the underlying actin cytoskeleton [18, 48]. Thus, the lack of Shank3 may destabilise receptor complexes expressed at glutamatergic synapses. Furthermore, the absence of Shank3 in MSNs has been shown to decrease the concentration of synaptic scaffolding proteins such as Homer, SAPAP, PSD93, and PSD95, and decrease the expression of glutamatergic receptor subunits GluA2, GluN1, GluN2A, and GluN2B [45, 50, 65, 73]. The reduction of these synaptic proteins in MSNs and the subsequent destabilisation of receptor complexes likely underlies the deficits in glutamatergic synaptic transmission detected in the cortico-striatal pathway of adult *Shank3*^*−/−*^ mice.

Maternal zinc supplementation had a differential effect on AMPAR versus NMDAR-mediated EPSCs in the dorsolateral striatal MSNs in adult offspring mice. In vitro experiments have demonstrated that Shank3 is necessary for the zinc-sensitive enhancement of AMPAR-mediated synaptic transmission [4, 27]. However, in the absence of Shank3, maternal zinc supplementation caused a further decrease in AMPAR-mediated EPSC amplitudes in adult *Shank3*^*−/−*^ mice in addition to the decrease in AMPAR EPSC amplitudes observed in adult *Shank3*^*−/−*^ mice in the control maternal zinc diet group. Therefore, overall there is a general inhibitory effect of maternal dietary zinc levels on AMPAR function independent of genotype. This indicates that zinc supplementation, from the beginning of brain development or post-weaning, (1) does not appear to utilise zinc-dependent regulation of AMPAR function as a mechanism underpinning ASD behavioural rescue as the decrease in AMPAR EPSCs in *Shank3*^*−/−*^ offspring mice is not reversed by maternal dietary zinc, and (2) is unable to prevent deficits in AMPAR-mediated synaptic transmission caused by the lack of *Shank3* at cortico-striatal synapses.

In contrast, maternal dietary zinc supplementation increased NMDAR-mediated EPSC amplitude at cortico-striatal synapses in adult *Shank3*^*−/−*^ mice to levels comparable with *Shank3*-wildtype mice from mothers that were fed with the control zinc diet. It must be noted however that maternal zinc supplementation also induced a significant decrease in NMDAR-mediated EPSC amplitudes in wildtype mice. As these animals displayed normal sociability, anxiety behaviours and lacked repetitive behaviours, this does not support a critical role of maternal dietary zinc-dependent increases in NMDARs as a primary mechanism underpinning a lack of ASD-related behaviour. However, given the major role that Shank proteins play in regulating postsynaptic glutamatergic receptor complexes, such upregulation of NMDAR function is very likely linked at least indirectly to the observed behavioural effects, especially given its important role in the induction of striatal synaptic plasticity. As occurs in response to direct zinc application [4], and dietary zinc supplementation to post-weaned animals [21], maternal zinc supplementation may be recruiting Shank2 to Shank3-lacking synapses in the offspring mice, and stabilising synaptic NMDARs by crosslinking NMDAR/PSD95 complexes with the underlying actin cytoskeleton. It is therefore plausible that key zinc-mediated synaptic alterations that occurred early during brain development allowed for the prevention of repetitive grooming behaviours in adult *Shank3*^*−/−*^ mice.

As the PPR can reflect altered presynaptic function, i.e., the probability of neurotransmitter release [8, 34, 43, 74], another possible mechanism through which maternal zinc supplementation could influence neuronal circuits and subsequently ASD-related behaviours is by restoring presynaptic function in *Shank3*^*−/−*^ mice via zinc-binding proteins in the presynaptic compartment. We observed that supplemental maternal dietary zinc reversed the decrease in PPR observed in *Shank3*^*−/−*^ mice, suggesting a return of release probability to that observed in wildtype mice. Several proteins that are involved in presynaptic vesicular transport and release are zinc-binding proteins, such as Bassoon and Piccolo [17, 20]. Zinc-dependent alteration of these proteins to regulate presynaptic vesicular release probability could prevent impaired PPR in adult *Shank3*^*−/−*^ mice from mothers that were fed with the supplemented zinc diet. In contrast, Peça et al. [50] did not observe deficits in presynaptic function in *Shank3*^*−/−*^ mice. However, the details regarding the dietary zinc levels fed to the *Shank3*^*−/−*^ mice in their study are unclear. “Control” diets sold commercially have significantly variable zinc concentrations [21]. Data from the present study and Fourie et al. [21] clearly demonstrate that dietary zinc supplementation can alter ASD-associated phenotypes from the synaptic to the behavioural level, and large differences between the zinc concentrations of rodent chow may contribute to variation between synaptic and behavioural phenotypes observed in mouse models of ASD.

The cortico-striatal pathway is only one of many networks in the brain implicated in ASD [2, 25, 38]. Changes to other brain regions have also been implicated in ASD, such as the hippocampus, prefrontal cortex, and amygdala. Studies investigating other *Shank3* deletion models of ASD have reported hippocampal deficits including reduced AMPAR-mediated synaptic transmission, decreased mEPSC amplitude and frequency, decreased mIPSC frequency, and impaired LTP [10, 33, 36, 40, 59, 66]. Additionally, reduced mEPSC and mIPSC frequencies have been reported in the prefrontal cortex of *Shank3* knockout mice [41, 73]. Other mouse models of ASD have reported deficits in many other regions such as the amygdala, cerebellum, and the anterior cingulate gyrus [26, 30, 41, 51]. As with the cortico-striatal pathway, it is likely that zinc supplementation alters glutamatergic synapses in these other regions. Therefore, examining the influence of maternal zinc supplementation on synaptic activity and structure in these regions will be important to provide a broader understanding of the mechanisms of zinc supplementation from the beginning of brain development in reversing ASD-associated behaviours. Moreover, simultaneously recording and correlating neuronal physiology with behaviour in awake, freely behaving ASD mice using in vivo imaging technology such as implantable miniaturised microscopes [1, 12], are critical in future studies to enable temporal correlation between specific ASD behavioural phenotypes and cellular changes in vivo.

How changes in the maternal level of dietary zinc are transferred to affect the pups in utero and postnatally is currently still not known. Zinc transporters have been shown to be expressed in rodent placentas allowing for zinc to transfer from the maternal circulation to the offspring [5, 44]. We were unfortunately unable to assess zinc transfer to the offspring through lactation, as separation of the *Shank3*^*+/−*^ mothers from their pups inhibited milk production and prevented us from obtaining any samples. We subsequently measured zinc levels in whole-brain samples from offspring born from mothers fed the control and supplemented zinc diets using ICP-MS. There was no genotype or maternal zinc supplementation-specific significant differences in the whole-brain zinc levels. However, several reasons could underlie this finding: a) ICP-MS provides a measure of total zinc levels without taking into account variations in zinc levels within the different subcellular compartments in glutamatergic neurons; b) measuring whole-brain zinc levels did not differentiate between potential variations in zinc levels between different regions of the brain, c) measuring brain zinc levels does not account for changes in the expression of zinc transporters that could have occurred as a result of *Shank3*-knockout or maternal zinc supplementation. Additionally, the effects of zinc on metabolism are unknown and beyond the scope of this study. Therefore, further studies are necessary to decipher how maternal zinc supplementation alters the level of zinc and zinc transporters in the brains of *Shank3*-wildtype and *Shank3*^*−/−*^ offspring.

In conclusion, we have revealed that maternal zinc supplementation from the beginning of brain development can prevent ASD-associated deficits in *Shank3*^*−/−*^ offspring mice that persist into adulthood. Collectively, these data highlight that maternal dietary zinc levels during pregnancy and lactation can alter neuronal synaptic function and ASD-associated behaviours in offspring. The replication of dietary zinc supplementation-induced prevention of ASD-associated behaviours in different genetic models and large animal models of ASD are now a critical next step to determine if maternal dietary or juvenile zinc supplementation is a potential preventative strategy in families with a history of heritable ASD.

## Supplementary information

**Additional file 1: Supplementary Figure 1.** Effect of control and supplemented zinc diet on the *Shank3+/-* breeders and the Shank3-WT and *Shank3-/-* offspring. **Supplementary Figure 2.** Measurements of zinc levels in dietary pellets and whole brain samples. **Supplementary Table 1.** Research Diets Inc, 30ppm and 150ppm zinc diet composition.

## Data Availability

All data generated or analysed during this study are included in this published article.
