# Supplementary material for: Influence of maternal zinc supplementation on the development of autism-associated behavioural and synaptic deficits in offspring Shank3-knockout mice
Source: Mol Brain. 2020 Aug 5;13:110. doi: 10.1186/s13041-020-00650-0 (PMC7409418; doi:10.1186/s13041-020-00650-0)
Supplement: Supplementary file 1 — Additional file 1: Supplementary Figure 1. Effect of control and supplemented zinc diet on the Shank3+/- breeders and the Shank3-WT and Shank3-/- offspring. Supplementary Figure 2. Measurements of zinc levels in dietary pellets and whole brain samples. Supplementary Table 1. Research Diets Inc, 30ppm and 150ppm zinc diet composition. [file 13041_2020_650_MOESM1_ESM.pdf]

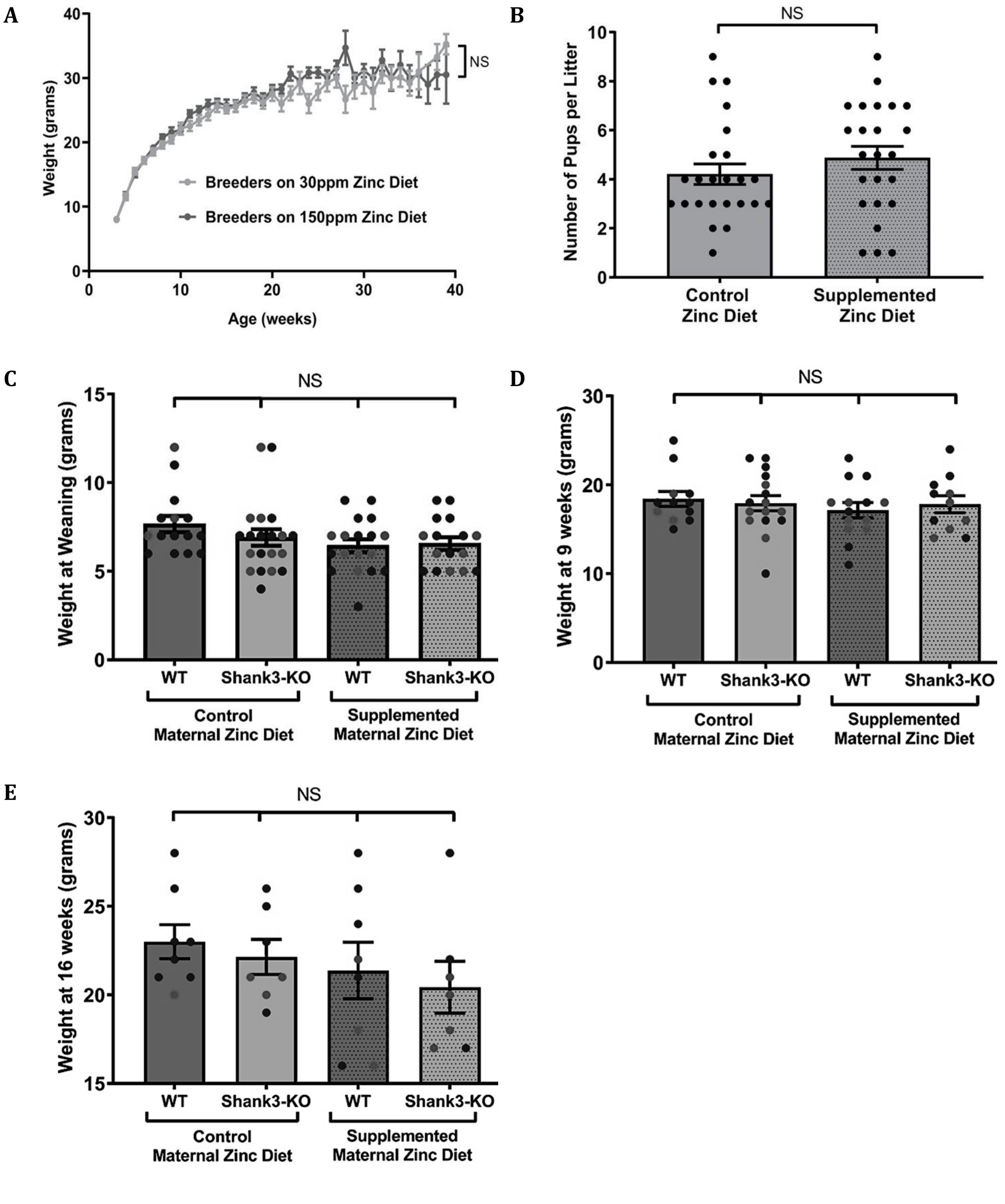

Supplementary Figure 1: Effect of control and supplemented zinc diet on the *Shank3*$^{+/-}$ breeders and the Shank3-WT and *Shank3*$^{-/-}$ offspring

(A) Weight of *Shank3*$^{+/-}$ breeders. No significant difference was observed in the weight of breeders fed a control (30ppm) or supplemented (150ppm) zinc diet from age of weaning (3 weeks) through development. **(B)** Number of pups born per litter. No significant difference was observed between the mean number of pups born per litter from mothers fed with the control versus mothers fed with the supplemented zinc diet. Weight of *Shank3*-wildtype (WT) and *Shank3*$^{-/-}$ (Shank3-KO) offspring from control MZD and supplemented zinc fed breeders is shown at **(C)** 3 weeks, **(D)** 9 weeks, and **(E)** 16 weeks of age. No significant differences were noted between these groups. All values are presented as

mean ± standard error of the mean. Individual data points represent individual animals, and were statistically analysed using two-tailed unpaired student's *t*-tests (A, B), or two-way ANOVA with Tukey's multiple comparisons test (C, D, E). NS = not significant.

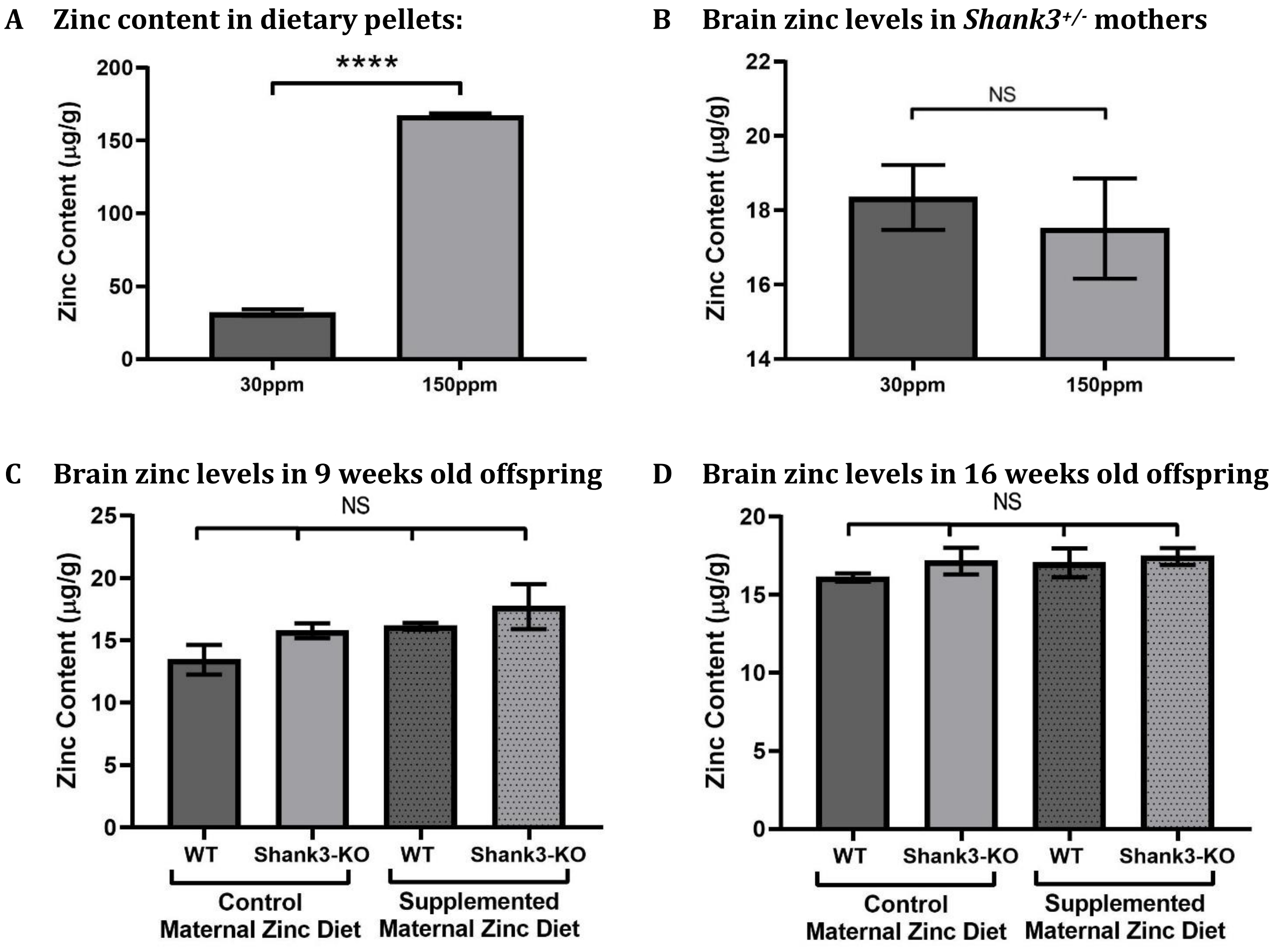

Supplementary Figure 2: Measurements of zinc levels in dietary pellets and whole brain samples

Dietary zinc levels were measured using inductively coupled plasma mass spectrometry. **(A)** Zinc levels are significantly higher in the supplemented (150ppm) dietary zinc pellets in comparison to the control (30ppm) dietary zinc pellets. **(B)** Whole-brain zinc levels were not significantly different between *Shank3*$^{+/-}$ mothers fed 30ppm or 150ppm zinc dietary. Whole-brain zinc levels were also not significantly different between wildtype (WT) and *Shank3*$^{-/-}$ offspring at 9 weeks of age **(C)**, or 16 weeks of age **(D)**. N=3 animals in each group, and were statistically analysed using two-tailed unpaired student's *t*-tests (A, B), or two-way ANOVA with Tukey's multiple comparisons test (C, D). NS = not significant, **** p<0.0001.

Supplementary Table 1: Research Diets Inc, 30ppm and 150ppm zinc diet composition

Product #	30ppm Zinc Diet (D19410B)		150ppm Zinc Diet (D06041101)	
	**gm%**	**kcal%**	**gm%**	**kcal%**
Protein	20.0	21	20.0	21
Carbohydrate	66.2	68	66.2	68
Fat	5.0	12	5.0	12
Total		100		100
**Ingredients**	**gm**	**kcal**	**gm**	**kcal**
Egg whites, dried	200	800	200	800
Corn starch	150	600	150	600
Sucrose	502.3795	2010	502.3795	2010
Cellulose, BW200	50	0	50	0
Corn oil	50	450	50	450
Mineral Mix S19407A	17.5	0	17.5	0
Calcium Phosphate, dibasic	17.5	0	17.5	0
Copper Carbonate	0.0105	0	0.0105	0
Ferric Citrate	0.21	0	0.21	0
Zinc Carbonate, 52.1% Zinc	0.056	0	0.286	0
Vitamin Mix V10001	10	40	10	40
Biotin, 1%	0.4	0	0.4	0
Choline Bitartrate	2	0	2	0
Pure FD&C Blue 1 Dye	0.1	0	0	0
Pure FD&C Red 40 Dye	0	0	0.1	0
**Total**	**1000.156**	**3900**	**1000.386**	**3900**
